# Burden of neck pain in general population of China, 1990–2019: An analysis for the Global Burden of Disease Study 2019

**DOI:** 10.7189/jogh.14.04066

**Published:** 2024-04-05

**Authors:** Weiwei Xia, Jiangmei Liu, Chenjun Liu, Shuai Xu, Kaifeng Wang, Zhenqi Zhu, Weiyan Wang, Huimin Wang, Haiying Liu, Maigeng Zhou

**Affiliations:** 1 Department of Spinal Surgery, Peking University People’s Hospital, Peking University, Beijing, China; 2 National Center for Chronic and Noncommunicable Disease Control and Prevention, Chinese Center for Disease Control and Prevention, Beijing, China; 3 Chinese Preventive Medicine Association, Committee on Prevention and Control of Spinal Disease, Beijing, China

## Abstract

**Background:**

Neck pain has become very common in China and has greatly affected individuals, families, and society in general. In this study, we aimed to report on the rates and trends of the prevalence, incidence, and years lived with disability (YLDs) caused by neck pain in the general population of China from 1990 to 2019.

**Methods:**

We used data from the Global Burden of Diseases, Injuries, and Risk Factors Study 2019 (GBD 2019) study to estimate the number and age standardised rates per 100 000 population of neck pain point prevalence, annual incidence, and YLDs in 33 provinces/municipalities/autonomous regions of China, stratified by age, sex, and sociodemographic index (SDI) from 1990 to 2019. We then compared these estimates with other G20 countries.

**Results:**

There were 6.80 × 10^7^ patients with neck pain in 2019, presenting an increase from 3.79 × 10^7^ in 1990. Likewise, the national age-standardised point prevalence increased slightly from 3.53% in 1990 to 3.57% in 2019. The YLDs increased by 78.08%, from 3814 × 10^3^ in 1990 to 6792 × 10^3^ in 2019. The age-standardised YLDs rate increased 1.50% from 352.84 in 1990 to 358.10 in 2019. The point prevalence of neck pain in 2019 was higher in females compared with males. These estimates were all above the global average level and increased more rapidly among G20 countries from 1990 to 2019. We generally observed a positive association between age-standardised YLD rates for neck pain and SDI, suggesting the burden is higher at higher sociodemographic indices.

**Conclusions:**

Neck pain is a serious public health problem in the general population in China, especially in its central and western regions, with an overall increasing trend in the last three decades. This is possibly related to changes of people's lifestyles and work patterns due to improvements in societal well-being and technology. Raising awareness of risk factors for neck pain in the general population and establishing effective preventive and treatment strategies could help reduce the future burden of neck disorders.

Neck pain is a very common symptom globally, especially in middle-aged and elderly people [1–4]. The findings of the Global Burden of Disease Study 2017 (GBD 2017) highlighted its high global prevalence and determined it as the leading cause of the burden of disability, especially in highly developed countries [1].

China encompasses a vast territory and a large population; consequently, its natural resources and economic development vary greatly between regions. Specifically, as the eastern region continued to develop economically due to governmental support after the 1990s, the central, western and northeastern regions began to follow. With the rapid improvements in technology and economy in recent decades, people’s working and living habits have also greatly changed. For example, the proportion of people doing white-collar work and those overusing smartphones has significantly increased, while the time for physical exercise and outdoor activities has substantially decreased [1]. In China, people now spend much longer time using smartphones, e.g. for entertainment, shopping, communication, and access to various services. This led to an increase in the number of cases of neck pain and a shift in the burden towards the younger population [1]. Neck pain not only brings physical and mental discomfort to patients and their families, seriously affecting their quality of life and work efficiency, but also unnecessarily burdens society’s medical resources [2,3,5]. Due to these factors, neck pain has become a serious health problem worldwide [1,6].

A few regional, epidemiological studies based on small sample sizes in China have shown that neck pain seriously affects the health of adolescents and adults [3,7–9]. However, they did not conduct stratified analyses on the burden in different regions with different development statuses; similarly, more recent data on neck pain have not been analysed for changes following the GBD 2017 study. According to the key indicators from China’s seventh national population census in 2021, China’s population has grown to more than 1.4 billion, with a greater proportion of elderly people than before due to constant demographic changes [10]. These shifts thus necessitate updated estimates for the burden of neck pain. In relation to this, the Global Burden of Diseases, Injuries, and Risk Factors Study 2019 (GBD 2019) provides new data and updated global and national assessments of the epidemiological characteristics of major diseases, including the prevalence, incidence, and years lived with disability (YLDs) caused by neck pain and their changes from 1990 to 2019 [11]. Further analysis of these data would help us better understand the current burden and impact of neck pain in China and its diverse regions at different development stages, which could in turn serve as a basis for aetiological studies and health care evaluation. To provide such insights, we aimed to estimate the incidence, prevalence. and years lived with disability (YLD) of neck pain in general population in China from 1990 to 2019, stratified by age, sex, provinces, and sociodemographic index (SDI) (a composite of sociodemographic factors). Our findings could help health policymakers understand the status of neck pain in order to establish timely and efficient prevention and control strategies.

## METHODS

### Overview

The GBD 2019 study provides a comprehensive assessment of all-cause and cause-specific mortality, as well as disability for all major diseases and injuries for 204 countries from 1990 to 2019, stratified by age, sex, and other demographic factors [11]. We focussed on the prevalence, incidence, and YLDs of neck pain in China.

### Definitions and data sources

GBD 2019 defined neck pain (ICD-10 code: M54.2, ICD-9 code: 723.1) as pain in the cervical spine region (with or without pain referred into the upper limbs) that lasts for at least one day [11,12].

The GBD 2019 derived data for China from national censuses, disease surveillance point systems, death cause registration report information systems, demographic surveys, and systematic reviews of published data. The study team developed the DisMod-MR 2.1 Bayesian meta-regression method to estimate the point prevalence, incidence, and YLD outcomes by different age and sex groups in all provinces of China, as detailed elsewhere [11]. In this study, we used disability weights (DWs) to represent the magnitude of health loss associated with neck pain, referring to those used in the GBD 2019 study measured on a scale from 0 (state of full health) to 1 (death) [13].

The GBD study developed four severity levels to describe the different levels of neck pain severity and its associated functional loss, as follows [11]:

− Mild: This person has neck pain and faces difficulty turning their head and lifting things. The DW is 0.052 (95% confidence interval (CI) = 0.036, 0.074).
− Moderate: This person has constant neck pain and faces difficulty turning their head, holding their arms up, and lifting things. The DW is 0.112 (95% CI = 0.079, 0.162).
− Severe: This person has severe neck pain and faces difficulty turning their head and lifting things. They get headaches and arm pain, sleep poorly, and feel tired and worried. The DW is 0.226 (95% CI = 0.147, 0.323).
− Most severe: This person has constant neck pain and arm pain, as well as difficulty turning their head, holding their arms up, and lifting things. They get headaches, sleep poorly, and feel tired and worried. The DW is 0.300 (95%CI = 0.199, 0.434).

### Data analysis

We analysed data on the prevalence, incidence, and YLD of neck pain from 1990 to 2019 for 33 provinces/regions, including 31 mainland provinces, municipalities, autonomous regions, and the Hong Kong and Macao Special Administrative Regions (SAR). We then selected 27 countries to compare with China: Argentina, Australia, Brazil, Canada, China, France, Germany, India, Indonesia, Italy, Japan, Mexico, Republic of Korea, Russian Federation, Saudi Arabia, South Africa, Turkey, United Kingdom, USA, Denmark, Finland, Norway, Sweden, South Sudan, Djibouti, Eritrea, and Burundi. These included nineteen G20 countries (grouped by their total gross domestic product (GDP) accounting for about 90% of the world), four Scandinavian countries, and three African countries. In the previous GBD 2017 study, neck pain was shown to be highly prevalent in Scandinavian countries and lowest in African countries [1].

We standardised the point prevalence and YLD rate by the constituent ratio of age of world population estimated by the GBD 2019 study [11]. We then calculated uncertainty intervals (UIs) from the standard errors generated from the input data, with ranges bounded by the 2.5 and 97.5 centile values, and the UIs were calculated from all steps of data manipulations.

We also conducted analyses stratified by SDI, a composite indicator of lagdistributed income per capita which can range from 0 (less developed) to 1 (most developed); the indicator considers gross domestic product per capita that has been smoothed over the preceding 10 years; average years of schooling in individuals >15 years old; and total fertility rates in females <25 years old [14]. We also used smoothing spline models to determine the association between the age-standardised YLD rate and SDI for 33 Chinese provinces from 1990 to 2019 [15].

## RESULTS

### National prevalence and incidence

**Figure 1:**
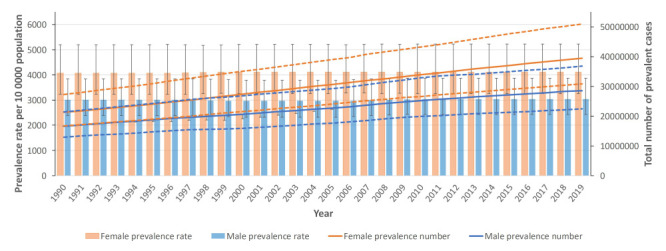
Age-standardised point prevalence (per 100 000 population) and number of prevalent cases of females and males in China from 1990 to 2019. Dotted lines indicate 95% upper and lower UIs.

The national age-standardised point prevalence of neck pain (from 0 to 100 years of age) in China was 3.57% (95% UI = 2.87, 4.52) in 2019, without a significant difference compared with 1990 (3.53%; 95% UI = 2.80, 4.49) (Table S1 in the **Online Supplementary Document**). There were 3.79 × 10^7^ (95% UI = 2.99, 4.86) individuals with neck pain in 1990; this number significantly increased to 6.80 × 10^7^ (95% UI = 5.37, 8.72) in 2019. Meanwhile we found no significant difference for age-standardised point prevalence of between these two study time point for females (1990: 4.07%, 95% UI = 3.23, 5.19 vs 2019: 4.11%, 95% UI = 3.26, 5.22) or males (1990: 3.02%, 95% UI = 2.39, 3.83 vs 2019: 3.03%, 95% UI = 2.42, 3.87). Although females had higher age-standardised point prevalence than males in both 1990 and 2019, this difference was likewise not significant (**Figure 1**).

The age-standardised annual incidences of neck pain per 100 000 population increased from 800.68 (95% UI = 634.43, 1016.63) in 1990 to 809.91 (95% UI = 641.89, 1028.61) in 2019, without significant differences. The incident cases of all ages with neck pain were 8.93 million (95% UI = 7.01, 11.43) individuals in 1990, increasing significantly to 14.89 million (95% UI = 11.67, 19.18) in 2019. We found no significant differences in incidence when comparing males or for females in 1990 vs 2019, or when comparing males and females to one another in either study time point (Figure S1 and Table S1 in the **Online Supplementary Document**).

**Figure 2:**
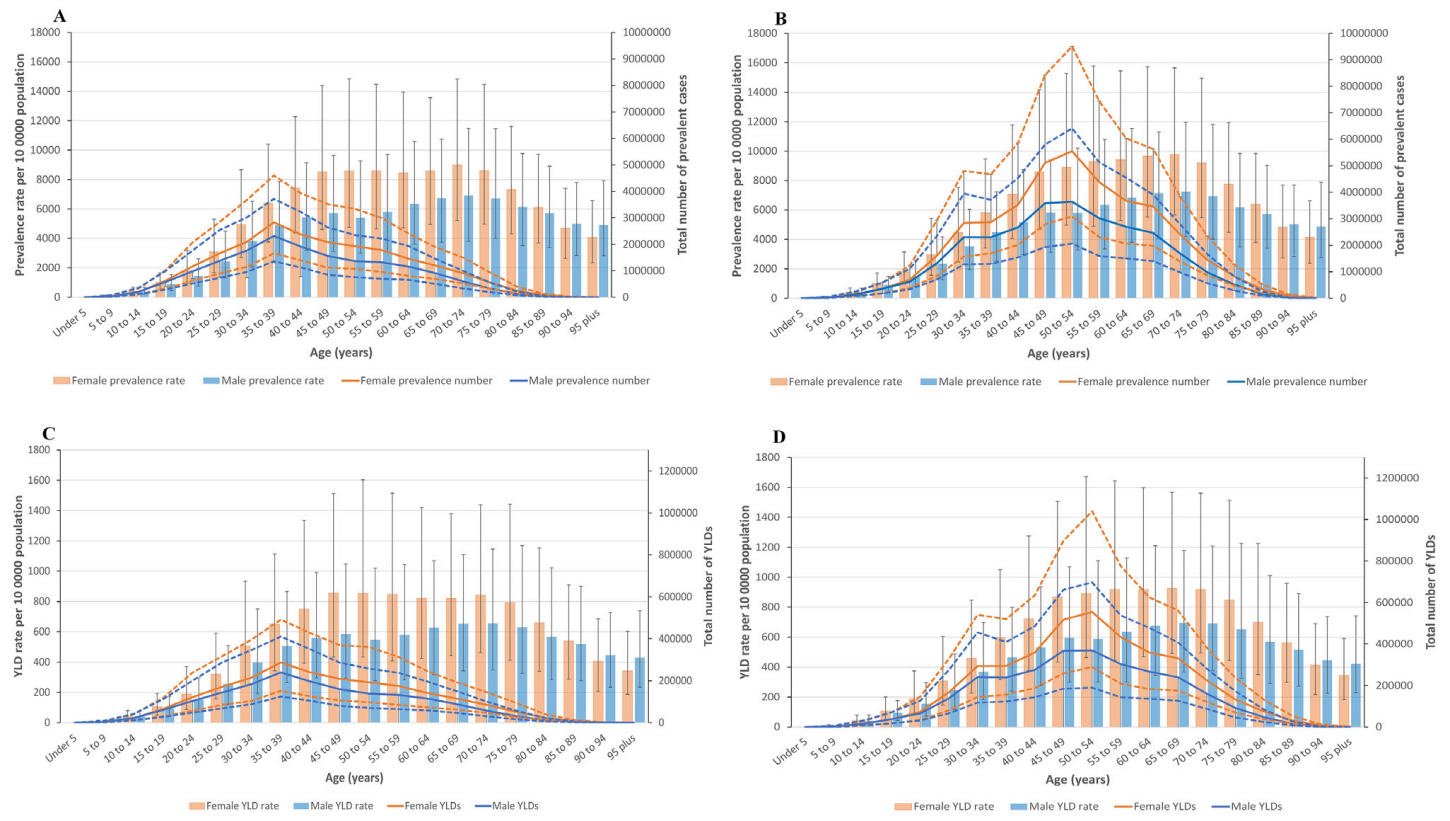
Age-standardised point prevalence (per 100 000 population) and number of prevalent cases by age and sex, 1990 and 2019, as well as age-standardised YLD rate (per 100 000 population) and number of YLDs by age and sex in 1990 and 2019. Dotted lines indicate 95% upper and lower UIs. **Panel A** **and** **Panel C.** 1990. **Panel B** **and** **Panel D**. 2019.

Furthermore, the national age-standardised point prevalence of neck pain initially increased from the age group 10–14 ages up to age group 70–74 years, then decreased with older age in both 1990 and 2019. We observed this trend for both sexes. We found no significant differences between males and females for age-standardised point prevalence across the age groups in both 1990 and 2019. Meanwhile, the prevalence in1990 and 2019 increased up to the 35–39 and 50–54-year-old age groups, respectively, then decreased with older age. The incidence of neck pain peaked in the 45–49-year-old age group in females and 60–64-year-old age group in males in 1990 and 2019, while the incident cases peaked in the 35–39 and 50–54-year-old age group for females and males in 1990 and 2019, respectively (**Figure 2**, Panels A–B; Figure S2 in the **Online Supplementary Document**).

### National YLD

The YLDs for neck pain in the general population increased by 78.08% (95% UI = 59.60, 98.31) from 3814.00 × 10^3^ (95% UI = 2479.01, 5573.88) in 1990 to 6792.00 × 10^3^ (95% UI = 4412.00, 9788.05) in 2019. The YLDs increased from ages of 10–14, and up to the peak at ages of 35–39 in 1990 and 50–54 in 2019, then decreased. The age-standardised YLDs rate per 100 000 population increased by 1.50% from 352.84 (95% UI = 230.02, 516.83) in 1990 to 358.1 (95% UI = 234.54, 515.95) in 2019, without a significant difference between the two time points. We likewise found no differences for males (0.71% increase (1990: 304.41; 95% UI = 194.97, 442.74 vs 2019: 306.58; 95% UI = 197.70, 444.75)) or females (1.49% increase (1990: 404.3; 95% UI = 267.10, 581.09 vs 2019: 410.32; 95% UI = 271.35, 585.92)) between the two study periods (**Figure 2**, Panels C–D and **Table 1**; Figure S3 in the **Online Supplementary Document**).

**Table 1:** Prevalence, incidence, and YLDs from neck pain in the general population in 1990 and 2019 for males and females, and percentage change of age-standardised rates per 100 000 population between 1990 and 2019*

	Male			Female			Both		
	**1990**	**2019**	**Change**†	**1990**	**2019**	**Change**†	**1990**	**2019**	**Change**†
**Prevalence, number ×100 000 (95% UI)**	166.01 (129.50, 213.84)	285.77 (223.91, 367.49)	72.14 (51.18, 93.43)	212.49 (167.42, 272.54)	393.89 (308.13, 509.46)	85.36 (64.35, 107.94)	378.51 (298.73, 485.58)	679.66 (536.73, 871.61)	79.56 (61.03, 99.70)
**Prevalence rate (95% UI)**‡	3015.70 (2392.36, 3833.04	3034.84 (2422.32, 3868.49)	0.63 (−6.68, 8.18)	4070.76 (3225.95, 5193.17)	4114.20 (3259.23, 5223.52)	1.07 (−6.33, 8.45)	3528.35 (2800.49, 4485.74)	3571.96 (2868.20, 4517.63)	1.24 (−4.53, 7.02)
**Incidence, number ×100 000 (95% UI)**	40.10 (31.46, 51.69)	64.61 (50.60, 83.01)	61.10 (44.13, 81.06)	49.20 (38.77, 63.03)	84.27 (65.60, 108.01)	71.27 (51.90, 92.67)	89.31 (70.08, 114.30)	148.88 (116.66, 191.84)	66.70 (49.56, 86.22)
**Incidence rate (95% UI)**‡	657.24 (515.64,847.15)	891.35 (698.16,1145.29)	0.50 (−4.16, 6.05)	857.97 (675.98, 1099.02)	1208.16 (940.49, 1548.53)	1.11 (−4.08, 5.84)	754.49 (592.06, 965.65)	1046.72 (820.22, 1348.75)	1.15 (−2.46, 5.09)
**YLD, number ×1000 (95% UI)**	1690.48 (1101.61, 2492.53)	2881.52 (1863.63,4211.54)	70.46 (50.10, 91.93)	2123.52 (1397.26, 3052.14)	3910.48 (2558.05, 5651.94)	84.15 (63.27, 106.33)	3814.00 (2479.01, 5573.88)	6792.00 (4412.00, 9788.05)	78.08 (59.60, 98.31)
**YLD rate (95% UI)**‡	304.41 (194.97, 442.74)	306.58 (197.70, 444.75)	0.71 (−6.54, 8.43)	404.30 (267.10, 581.09)	410.32 (271.35, 585.92)	1.49 (−5.83, 8.93)	352.84 (230.02, 516.83)	358.13 (234.54, 515.95)	1.50 (−4.27, 7.29)

UI – uncertainty interval, YLD – years lived with disability

*Lower UIs and upper UIs are the 2.5 to 97.5 centile values of the 95% UIs.

†Changes are the total mean values in 2019 compared with 1990.

‡Age-standardised prevalence, incidence, and YLD rate (per 100 000 persons).

### Provincial prevalence and YLD

**Figure 3:**
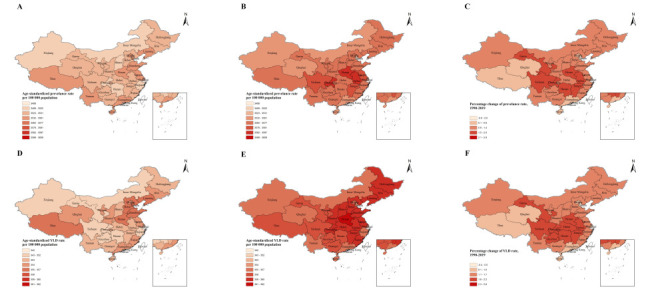
Age-standardised point prevalence and YLD rate (per 100 000 population) of 33 provinces/municipalities/autonomous regions in China, 1990 and 2019 and the changes between 1990 and 2019. **Panels A and D.** 1990. **Panel B and Panel E.** 2019. **Panel C and Panel F.** Changes between 1990 and 2019

Among the 33 provinces/municipalities/autonomous regions in China, we observed the highest age-standardised point prevalence of neck pain in Tibet in 1990 (3.56%; 95% UI = 2.83, 4.53) and Hong Kong in 2019 (3.61%; 95% UI = 2.84, 4.58). Overall, Hong Kong had lowest point prevalence of neck pain in 1990 (3.41%; 95% UI = 2.74, 4.31), while Tianjin had the lowest prevalence in 2019 (3.52%; 95% UI = 2.82, 4.45). From 1990 to 2019, Tianjin was the only city for which the age-standardised point prevalence decreased between the two study time points, even though the difference was not significant (−0.55%, 95% UI = −6.24, 4.91). The percentage changes of prevalence rate were higher in the central part of China, while Hong Kong had the greatest change with an increase of 5.88% (95% UI = −1.11, 13.51). The age-standardised point prevalence in most provinces/regions (n = 32) was below 3.55% of the point prevalence in 1990, with only slight disparity among different provinces/regions; most (n = 31) were above 3.55% in 2019 (**Figure 3****,** Panels A–C).

In 1990, the percentage of age-standardised YLDs caused by neck pain among the 33 provinces/municipalities/autonomous regions in China ranged from 3.47% to 3.93%. Furthermore, neck pain ranked as the fifth leading cause of YLDs in all diseases overall; it was the fifth leading cause in 13, the sixth leading cause in 13, the seventh leading cause in 6, the eighth leading cause in 1 of the 33 provinces/municipalities/autonomous regions. In 1990, Macao, Shandong, and Tibet had the highest age-standardised YLD rates at 355.73, 355.26, and 355.12 per 100 000 population, respectively, while Hong Kong, Hunan, and Zhejiang had the lowest at 342.27, 350.97, and 351.36 per 100 000 population.

In 2019, the percentage of age-standardised YLDs caused by neck pain ranged from 3.86% to 4.19% in 33 provinces/ regions in China, with neck pain remaining the fifth leading cause of YLDs among all diseases. Specifically, it was the fifth leading cause in 15, the sixth leading cause in 14, and the seventh leading cause in 4 of the 33 provinces/municipalities/autonomous regions in China. In 2019, Hong Kong, Macao, and Henan had the age-standardised YLD rates at 362.16, 362.00, and 360.91 per 100 000 population, respectively, while Tianjin, Hainan, and Guangdong had the lowest at 353.40, 355.28 and 355.79 per 100 000 population. From 1990 to 2019, Hong Kong, Chongqing, and Anhui had the three greatest increase of YLD rate at 5.8% (95% UI = −1.9, 1.4), 2.16% (95% UI = −3.94, 8.77) and 2.15% (95% UI = −4.10, 8.39), respectively. Tianjin was the only one city with a decreasing YLD rate of 0.43% (95% UI = −6.58, 6.02). Overall, the rank of neck pain increased from the seventh (1990) to the fifth (2019) leading cause of all-age YLDs (**Figure 3**, Panels D–F; Table S2 and Figure S4 in the **Online Supplementary Document**).

### National comparisons

In 2019, China had a high age-standardised point prevalence, ranking eight among the 27 countries. The USA, the UK, and Denmark had the highest age-standardised point prevalence, while Australia, Djibouti, and Burundi had the lowest. Furthermore, the USA, China, and South Sudan had the highest increases in age-standardised point prevalence between 1990 and 2019, while Norway, Burundi, and Republic of Korea had the lowest. Furthermore, China had an overall high age-standardised annual incidence in 2019, ranking fourth among the 27 countries, with Indonesia, the USA, and the UK having the highest and Australia, Djibouti, and Burundi having the lowest incidence. The USA, the UK and China had the highest increase from 1990 to 2019; Norway, Burundi, and Republic of Korea had the lowest (Table S1 in the **Online Supplementary Document**).

China also had an overall high YLD rate among the 27 countries, as it was higher than the average global level (267.35, 95% UI = 175.53, 383.54). The USA, the UK and Denmark had the highest YLD rate, while Australia, South Sudan and Burundi had the lowest. The USA, China and South Sudan had the highest increases from 1990 to 2019; Norway, Burundi, and Germany had the lowest (Table S1 in the **Online Supplementary Document**).

### SDI

**Figure 4:**
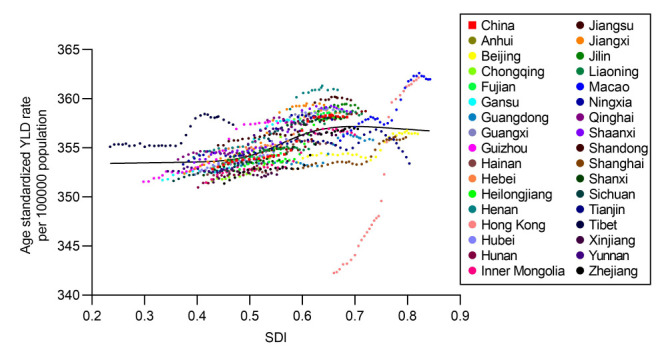
Age-standardised YLD rate (per 100 000 population) from neck pain for 33 provinces by SDI, 1990–2019.

Overall, we observed a positive association between the age-standardised YLD rate from neck pain and SDIs across 33 provinces/regions in China with fluctuations from 1990 to 2019, where the burden of neck pain is increased with higher levels of socioeconomic development. The burden of neck pain, however, was not always high in provinces with a high SDI at different time points from 1990 to 2019. For instance, the burden of neck pain in Tibet was much higher than the expected level based on its SDI, but while the burden of neck pain in Hongkong simultaneously lower than expected. Moreover, SDI gradually increased downward from the eastern towards the western regions in China both in 1990 and 2019. The change of SDI from 1990 to 2019, however, showed that most of the western and central regions had a higher SDI than the eastern regions (**Figure 4**; Figure S5 in the **Online Supplementary Document**).

## DISCUSSION

In this study, we reported on the prevalence, incidence, YLDs, and age-standardised rates for neck pain in the general population across 33 provinces/regions in China from 1990 to 2019. We observed that there were 67.97 million prevalent cases, 14.89 million incident cases, 6.79 million years lived with disability in 2019. Although the age-standardised rates of point prevalence, annual incidence, and YLDs from neck pain have not changed significantly over the study period, the number of prevalent cases, incident cases, and YLDs increased. The age category of peaked number of prevalence, incidence, and YLDs shifted from the 35–39-year-old age group in 1990 to 50–54-year-old age group in 2019. The possible reasons behind these changes could be due to the population growth and ageing.

Neck pain has become a public health challenge in many countries in recent years [1,6]. From the study of neck pain based on GBD 2017, China had one of the higher age-standardised annual incidence, point prevalence, and YLD rate among 195 countries [1]. Scandinavian countries, including Norway, Finland, Denmark and Sweden, were reported to have higher prevalence estimates than other countries [1,16]. Based on the the GDB 2019 results, USA and UK have overcome Scandinavian countries in terms of age-standardised prevalence, incidence, and YLD rate from the GBD 2017 study. China still remained above the global average, with a much higher increased rate change among the selected 27 countries in 2019 in our study.

The age-standardised point prevalence of neck pain in China was 3.57% (95% UI = 2.87, 4.52) in 2019, without a significant increase from 1990 (3.53%; 95% UI = 2.80, 4.49). This may be due to the changes of lifestyles and work patterns. Although the number of people engaged in heavy manual work such as agriculture has decreased, the number of people engaged in scientific and technological work has increased. The number of individuals with neck pain, however, significantly increased from 3.79 × 107 (95% UI = 2.99, 4.86) in 1990 to 6.80 × 107 (95% UI = 5.37, 8.72) in 2019, possibly due to population growth from 1.1 billion in 1990 to 1.4 billion in 2019. In China, the trend of point prevalence rate of neck pain in the last three decades differed from that of low back pain [17]. From 1990 to 2016, the overall point prevalence of low back pain has decreased, while it has not changed significantly for the neck pain. Therefore, lifestyle changes and prevention strategies in past decades may have effectively decreased the occurrence of low back pain. Nevertheless, neck pain was underemphasised and understudied by researchers, clinicians, and policymakers in recent decades.

We found a higher age-standardised point prevalence among women than men both in 1990 and 2019, which is consistent with other previous studies [1,18–21] and similar to previous findings that many forms of pain are more common in women than in men [17,22,23]. The potential mechanisms underlying these sex differences could be biological or psychosocial. One biological mechanism could be the gonadal hormones possibly causing a heightened inflammatory response and increased nociceptive afferents in periphery in women or the multiple central nervous system pathways on pain responses being influenced by sex differences of gonadal steroids, such as endogenous opioid systems, dopaminergic function, serotonergic activity [24,25]. Likewise, women and men may also differ in endogenous pain modulation, such as sex-specific brain areas of activation by pain stimuli, and there is also the more pronounced analgesia function of diffuse noxious inhibitory controls (DNIC) in males [26,27]. The psychosocial contributions include the differences in cognitive-emotional and behavioural factors associated with men and women’s pain. Some studies reported that affective factors and pain-related catastrophising have a stronger relationship with pain in women compared with men, such as more mental stress (eg, anxiety, depression) and coping processes; women with more intense pain have more coping strategies than men, such as using more social and emotional support [28,29]. According to published reports, women are less likely to be physically active than men and spend more time in sedentary activity, which leads to the increased experience of neck pain [30,31].

As disability-adjusted life years are the standard metric used to quantify burden of a disease, calculated by combining years of life lost due to premature mortality and YLDs, we used YLDs to quantify the burden of neck pain as there is no evidence of mortality from neck pain [1]. Although the age-standardised point prevalence rate of neck pain did not increase significantly (from 3.53% in 1990 to 3.57% in 2019), the population increasing and ageing contributed to the growth of YLDs. The YLDs for neck pain increased by 78.08% from 3814.00 × 10^3^ (95% UI = 2479.01, 5573.88) in 1990 to 6792.00 × 10^3^ (95% UI = 4412.00, 9788.05) in 2019. The age category of peaked YLDs shifted from the 35–39-year-old age group in 1990 to the 50–54-year-old age group in 2019. This is similar to and could likewise be explained by the changes of low back pain, which may be due to the longer life expectancy and the growth in the aged populations [17]. Another underlying reason could be the more effective medical strategies for treating neck/low back pain than before. Painfulness can be cued at early stage at younger age, but with the progression of spinal disease, the effect of pain treatment is reduced, such as the pain related to nerve injury [32]. Moreover, people’s education time became longer, thus, the age at which individuals first engage in work increased [33]. Based on the above-mentioned reasons, the age category with the peak YLDs shifted towards the older group in 2019 compared to 1990. The age-standardised YLDs rate per 100 000 population was 358.13 (95% UI = 234.54, 515.95) in 2019, which was higher than global level (267.35; 95% UI = 175.53, 383.54). The age-standardised YLDs rate in 2019 was not different from 1990, which is consistent with the global trend [1]. Moreover, we found YLDs and YLD rates of neck pain to be higher for females than males both in 1990 and 2019, but with no significant difference. Namely, the influence of neck pain on females and males were also similar.

Neck pain imposes an enormous personal and socioeconomic burden on society, with a prevalence approaching that of low back pain [34]. In terms of YLDs, neck pain ranked as the fifth disease in China in both 1990 and 2019, after low back pain, age-related hearing loss, headache disorders, and depressive disorders. In 2019, Hong Kong, Macao and Henan had the highest age-standardised YLD rates at 362.16, 362.00, and 360.91 per 100 000 population, respectively, presenting increases of 5.8%, 1.8%, and 1.9% from 342.29, 355.73, and 354.05 per 100 000 population in 1990, when the mean increased age-standardised YLDs rate of total China population between 1990 and 2019 was 1.5%. Hong Kong had the largest increase, probably due to rapid population increase and economy development, especially for the increased population of managers, administrators, and professionals [18]; Chongqing and Anhui had the second and third largest increase by 2.16% and 2.15%, respectively. Tianjin was the only one city with a decreasing YLD rate by 0.43% from 1990 to 2019, although this difference was not significant. Overall, neck pain is still considered as one of the main diseases causing YLDs. The rank of neck pain increased in all-age YLDs, which may result from the increasing of age-standardised point prevalence of neck pain from 1990 to 2019.

A previous study found a positive association between regional-level SDIs and age-standardised YLD rates for neck pain between 1990 and 2017 globally [1]. Our findings further confirmed this association. With societal development, the working and living styles came to differ between cities with higher and lower SDI. Several studies have demonstrated an association between psychosocial factors and musculoskeletal disorders [35–37]. In higher developed cities with more people of higher education level, the stiff competitive working environment, high stress at work, and long working hours may account for the high prevalence of neck pain, such as managers, administrators, and professionals [38].

With the development of the global technology and economy, people’s lifestyles have greatly changed. Heavy labour occupations have decreased due to the industry development. More people began to take the repetitive and precision work with sedentary work position (i.e. prolonged standing, sitting, or doing computer work), working with the cervical spine in ﬂexion for prolonged periods of time. Notably, people usually look down on the electric mobile devices, such as tablets or mobile phones, while walking on the street or using transportations [39]. The compensatory mechanisms in head-spine-limb musculoskeletal system play important roles in maintaining the global balance of human body, and the excessive and longtime of forwarded head posture can cause excessive curves of other body parts to maintain balance [40,41]. These conditions undoubtedly aggravate painfulness of the neck, shoulder, and back. In 2019, Hong Kong had the highest age-standardised point prevalence of neck pain (3.61%), with the highest increase of 5.88% compared to 1990 (3.41%). In the last three decades, neck pain has been found to be highly prevalent in Hong Kong particularly among subjects of a high socioeconomic status [8,18,42,43]. Before the 1990s, the eastern part of China was primarily developing industrially, while other parts of China focussed on agriculture; after the 1990s, the central, western and northeastern regions of China gradually began to develop as well. This also agrees with our finding that the change of SDI in western and central regions from 1990 to 2019 were higher than eastern regions, which further led to the percentage changes of prevalence and YLD rate being higher in the central and western parts of China from 1990 to 2019. Furthermore, we also found a higher prevalence of neck pain in developed industrial western countries compared to underdeveloped African countries, which are dominated by agriculture and animal husbandry. The lifestyles and work patterns between those countries in different developments are clearly different. The most affected jobs include continuous work with hands above the head or elbows above the shoulders, such as teachers and decoration workers; poor static or dynamic postural work, such as dentists and painters; long-term desk work, such as office and computer workers; high-intensity work causing stress on the cervical spine, such as pilots, competitive athletes, etc. The long working time and high stress at work in highly developed urban areas may contribute to the high prevalence of neck pain.

Improving people's awareness of taking physical activity is an important part of Healthy China 2030 plan [44]. Physical Activity Guidelines for Chinese People (2021) [45] and A Guide for the Prevention and Treatment of Work-related Musculoskeletal Disorders (2022) [46] provide detailed guidance on the types and time of exercises for different groups of people and strongly recommend that people try to reduce the sedentary behaviours, such as sitting quietly, watching TV or videos, and using computers. These behaviours cannot only lead to obesity and short-sightedness, but also may cause many musculoskeletal disorders, especially neck and back pain. The following preventive and control strategies are recommended for reducing neck pain: Relieve pessimistic anxiety and stress by diverting attention, especially for women, such as entertainment, sports, music, etc.; correct the wrong posture, avoid working with head down for a long time, sleeping in improper high pillows, and use simple neck medical gymnastics to relieve neck muscle fatigue; teachers and family members should remind children and adolescents with poor self-restraint to supervise their movements and studying habits; old people should exercise appropriately, avoiding long time of sedentary behaviours; employers should improve working conditions by avoiding long-term repetitive work and excessive physical load, helping workers master the correct way of working, carrying out reasonable exercise, and adapting work tasks to personal needs. Overall, the strategies to reduce neck pain prevalence requires multisectoral involvement, such as family, school, health care, work unit and government. Most importantly, there is an urgent need to raise people’s awareness of neck pain and have a healthy lifestyle.

Our study is subject to all the limitations of the previous GBD studies. First, GBD 2019 estimated the models of neck pain mainly through available provinciallevel data and previous literature in China. The absence of relevant data in some provinces and in some years might have led to bias in the model estimates, especially because there is still few epidemiological studies of neck pain in China. Therefore, it is necessary to continue to collect more detailed data for analysis and encourage more studies on neck pain epidemiology. Second, in the 2019 GBD study, there were many variations in recalling period, anatomical location, and minimum duration of episodes. We suggest future research uses standardised neck pain assessment method. Third, the YLD calculations rely on the disability weights in GBD 2019 study, which are mainly obtained from the survey of European and American populations. For the feelings of severity of neck pain, there are large differences among different races and cultural backgrounds – for example, between Chinese and European individuals. Therefore, there could have been some biases in the measurement of the burden of neck pain among Chinese population in GBD 2019. In future studies, the disability weight survey data from Chinese population can be added to make China's estimation more accurate [47]. Last, we analysed the status and trend of neck pain, but were unable to consider specific risk factors of neck pain due to the lack of related data; follow-up research needs to address this gap to better guide neck pain prevention efforts..

## CONCLUSIONS

Neck pain is a serious public health challenge in the general population in China, with the increasing trend in the last three decades. With the development of society, the advancement of science and technology, and the changes of people's lifestyles and work patterns, the burden of neck pain could increase in the future, especially in the central and western regions of China. Increasing population awareness about risk factors and preventive strategies for neck pain is warranted to reduce the future burden of neck disorders, such as cervical spondylosis.

## Additional material


Online Supplementary Document.
