# Supplementary material for: Burden of neck pain in general population of China, 1990–2019: An analysis for the Global Burden of Disease Study 2019
Source: J Glob Health. 2024 Apr 5;14:04066. doi: 10.7189/jogh.14.04066 (PMC10994671; doi:10.7189/jogh.14.04066)
Supplement: Online Supplementary Document. [file jogh-14-04066-s001.pdf]

Supplementary Figure 1 Age standardized incidence (per 100 000 population) and number of incident cases of females and males in China from 1990 to 2019. Dotted lines indicate 95% upper and lower uncertainty intervals.

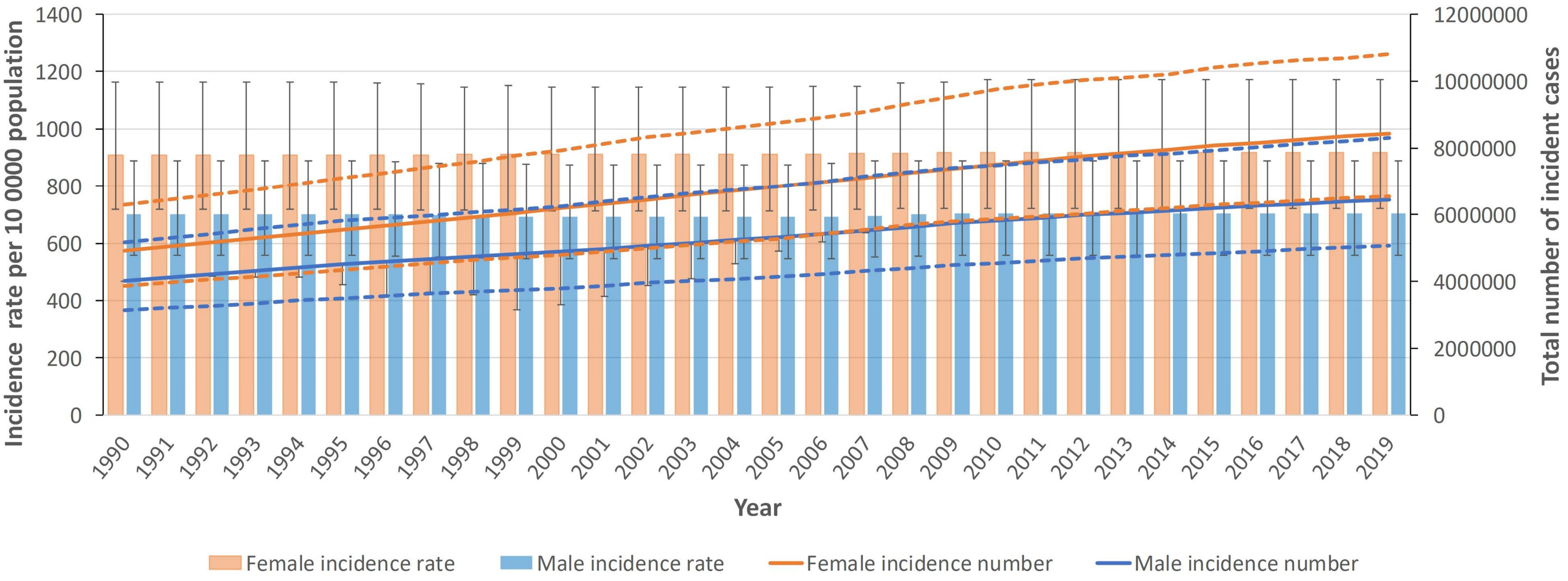

Supplementary Figure 2 Age-standardized incidence (per 100 000 population) and number of incident cases by age and sex in 1990 and 2019. Dotted lines indicate 95% upper and lower uncertainty intervals. A:1990; B:2019.

A

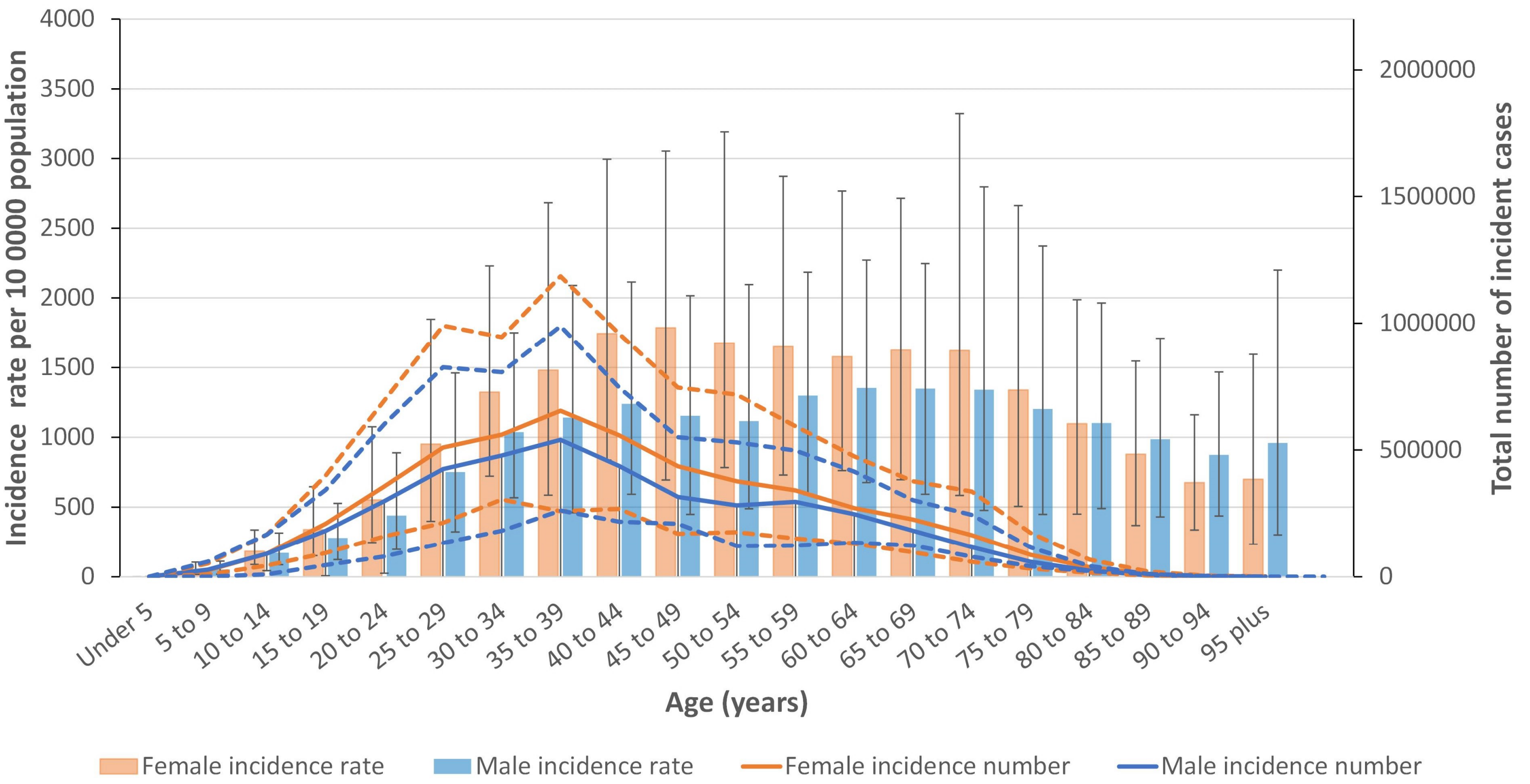

B

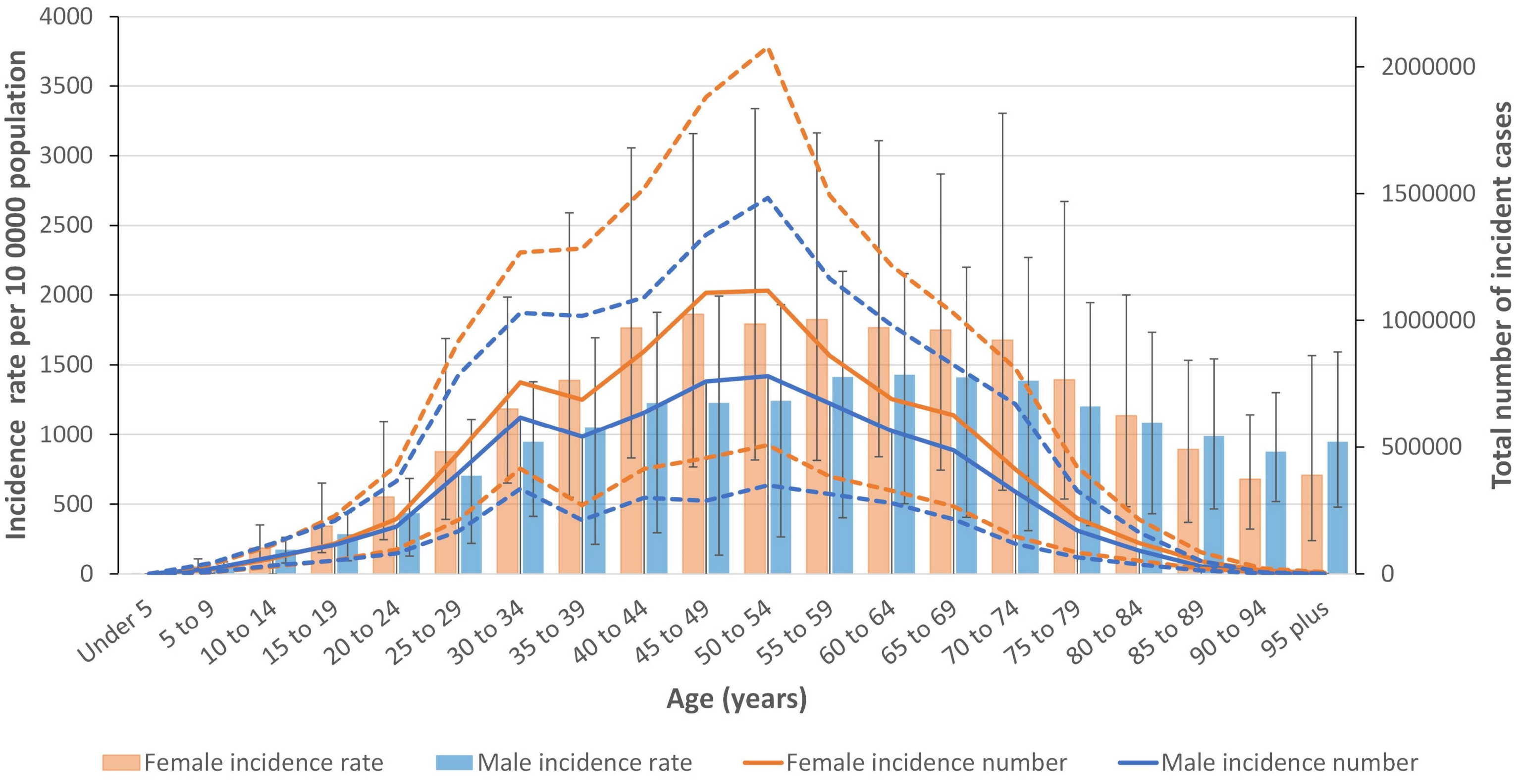

Supplementary Figure 3 Age standardized years lived with disability (YLD) rate (per 100 000 population) and number of YLDs of females and males in China from 1990 to 2019. Dotted lines indicate 95% upper and lower uncertainty intervals.

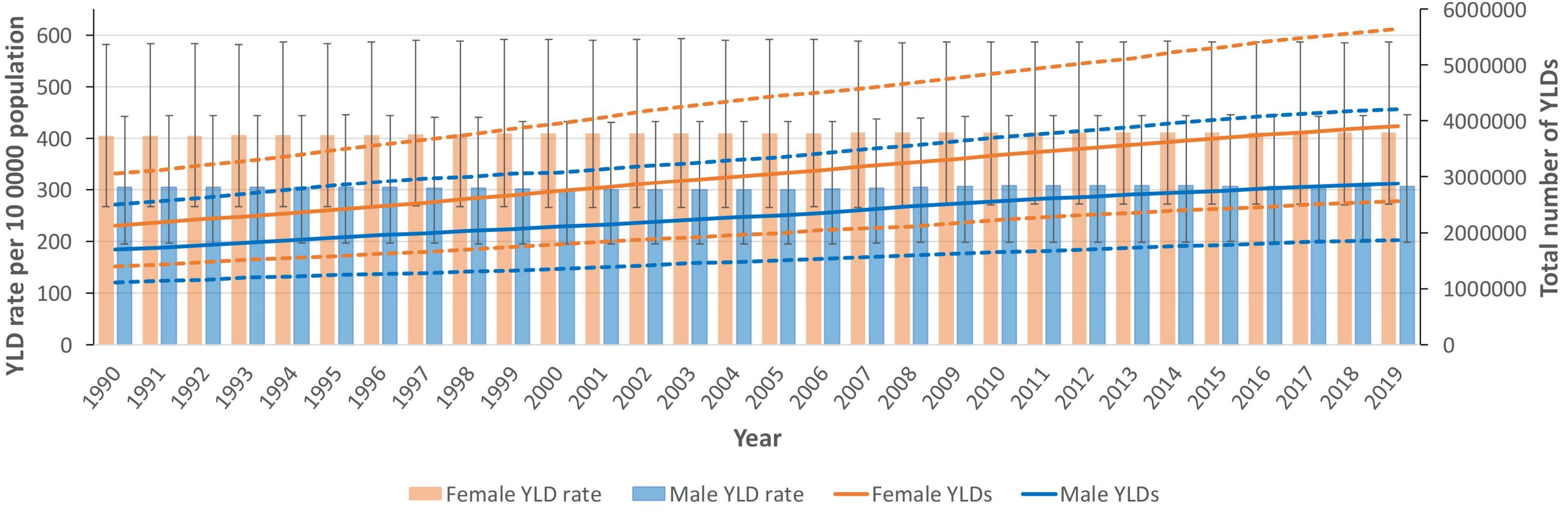

Supplementary Figure 4

The rank of major diseases by all ages and age-standardized years lived with disability (YLDs) rate (per 100 000 population) in 33 provinces/regions of China (1990 and 2019).

A

Both sexes, Age-standardized, 1990, YLDs per 100,000

	China	Anhui	Beijing	Chongqing	Fujian	Gansu	Guangdong	Guangxi	Guizhou	Hainan	Hebei	Heilongjiang	Henan	Hubei	Hunan	Inner Mongolia	Jiangsu	Jiangxi	Jilin	Liaoning	Ningxia	Qinghai	Shaanxi	Shandong	Shanghai	Shanxi	Sichuan	Tianjin	Tibet	Xinjiang	Yunnan	Zhejiang	Hong Kong	Macao
Low back pain	1	1	1	1	1	1	1	1	1	1	1	1	1	1	1	1	1	1	1	1	1	1	1	1	1	1	1	1	1	1	1	1	1	1
Age-related hearing loss	2	2	2	2	2	2	2	2	2	2	2	2	2	3	2	2	2	2	2	2	2	2	2	2	2	2	2	2	2	2	2	2	2	2
Depressive disorders	3	3	7	3	3	3	4	4	3	3	3	4	3	2	3	3	4	3	4	5	5	3	3	3	7	4	4	8	4	3	3	3	3	4
Headache disorders	4	4	3	4	4	4	3	3	4	4	4	3	4	4	5	4	3	4	3	3	4	4	4	4	3	3	3	3	3	4	4	4	4	3
Neck pain	5	6	6	6	5	6	5	5	6	7	5	7	5	6	6	5	7	5	6	6	6	5	5	7	6	6	7	5	7	5	6	8	5	5
Anxiety disorders	6	8	12	7	7	5	15	6	8	8	8	8	6	7	4	7	8	8	7	14	3	14	7	5	16	7	8	10	8	8	7	9	12	6
COPD	7	7	10	5	6	7	6	8	9	10	10	9	8	8	7	8	6	6	10	9	8	7	9	6	8	10	6	9	9	9	8	7	11	8
Gynecological diseases	8	9	5	8	9	8	7	7	5	9	7	10	9	9	8	9	9	9	9	7	7	6	8	8	4	11	5	6	11	10	5	10	8	9
Dietary iron deficiency	9	5	18	9	15	9	14	9	7	5	9	12	11	5	9	15	5	7	11	10	13	8	6	10	5	5	9	14	6	7	21	6	18	14
Stroke	10	12	4	15	13	15	12	11	13	13	6	5	7	11	13	6	15	12	5	4	9	12	14	9	12	8	18	4	10	14	13	16	14	10
Blindness and vision loss	11	11	13	10	11	10	8	10	15	11	11	11	10	10	10	10	11	10	12	15	10	10	10	13	19	15	15	15	5	13	9	12	9	13
Diabetes	12	10	11	12	10	11	9	14	11	6	12	6	14	15	11	12	16	13	8	8	11	13	13	15	14	9	12	7	14	6	11	14	16	18
Oral disorders	13	13	16	14	12	12	16	12	14	14	13	14	12	13	12	13	14	14	14	13	12	9	12	12	17	12	10	13	13	16	15	13	6	12
Other musculoskeletal	14	14	9	17	14	17	11	13	18	15	14	13	13	12	14	14	12	17	13	11	15	17	11	11	10	14	14	11	17	12	20	15	7	7
Endo/metab/blood/immune	15	16	8	16	17	18	10	15	19	16	16	15	15	14	16	16	13	18	15	12	14	16	16	16	9	16	13	12	15	17	17	11	10	11
Falls	16	15	14	11	8	20	17	16	17	12	20	18	18	17	17	22	10	15	17	18	18	15	17	17	11	17	17	17	12	18	16	5	13	16
Osteoarthritis	17	18	17	13	18	14	18	18	12	17	17	17	16	16	18	17	17	19	16	16	16	11	15	18	20	13	11	18	18	15	12	18	17	15
Schizophrenia	18	17	15	19	16	21	13	20	20	18	15	16	17	18	20	18	18	11	21	17	17	18	18	14	18	18	19	16	20	20	18	17	19	17
Drug use disorders	19	19	20	18	19	16	20	17	10	19	18	21	20	19	15	19	19	16	19	19	20	20	19	19	21	19	20	19	16	19	10	20	15	20
Dermatitis	20	20	22	20	21	22	23	21	23	21	19	20	19	20	19	21	21	20	20	20	19	21	20	20	13	21	22	20	22	21	23	21	20	21

B

Both sexes, Age-standardized, 2019, YLDs per 100,000

	China	Anhui	Beijing	Chongqing	Fujian	Gansu	Guangdong	Guangxi	Guizhou	Hainan	Hebei	Heilongjiang	Henan	Hubei	Hunan	Inner Mongolia	Jiangsu	Jiangxi	Jilin	Liaoning	Hong Kong	Macao	Ningxia	Qinghai	Shaanxi	Zhejiang	Yunnan	Shandong	Shanghai	Shanxi	Sichuan	Tianjin	Tibet	Xinjiang
Low back pain	1	1	1	1	1	1	1	1	1	1	1	2	1	2	1	1	1	1	2	1	1	1	1	1	2	4	2	2	3	1	1	1	2	1
Age-related hearing loss	2	2	2	2	2	2	2	2	2	2	2	3	2	1	2	2	2	2	1	2	2	2	2	2	1	1	1	1	1	2	2	2	1	2
Headache disorders	3	3	3	3	3	3	3	3	3	3	3	1	3	3	4	3	3	3	3	3	4	3	3	3	3	3	3	3	2	3	3	3	3	3
Depressive disorders	4	4	6	4	4	4	4	4	4	5	4	5	4	4	5	4	4	4	5	6	3	4	5	4	4	2	4	4	9	4	4	9	4	4
Neck pain	5	5	5	5	5	6	6	5	5	6	6	7	6	5	6	6	5	5	7	7	6	6	6	5	5	5	5	6	5	6	5	7	6	6
Other musculoskeletal	6	6	4	6	6	7	5	6	7	8	8	8	7	6	7	7	6	6	6	8	5	5	8	7	6	7	12	7	4	8	8	6	9	7
Diabetes	7	7	7	8	7	8	7	9	8	4	7	6	8	13	8	8	9	10	8	5	14	14	9	6	9	9	6	10	8	5	9	4	11	5
Stroke	8	8	8	13	12	10	9	7	6	7	5	4	5	10	9	5	7	13	4	4	15	9	7	10	8	16	8	8	13	7	13	5	7	10
Anxiety disorders	9	9	13	10	8	5	18	8	9	9	9	9	9	7	3	9	8	7	9	13	12	7	4	14	7	6	10	5	14	9	12	8	8	8
Gynecological diseases	10	10	9	9	14	12	10	11	10	12	10	11	11	9	10	12	10	14	10	9	8	8	10	9	14	10	7	14	6	13	7	11	17	12
Blindness and vision loss	11	11	14	12	13	11	11	10	15	10	11	10	10	8	12	10	14	9	11	14	9	16	12	13	12	15	9	15	18	14	17	13	5	14
COPD	12	13	21	7	16	9	14	12	11	15	15	14	14	11	11	11	13	11	16	17	18	15	13	8	16	17	11	12	22	16	6	17	10	9
Oral disorders	13	12	16	15	11	14	17	13	14	13	12	12	13	14	13	14	15	15	12	12	7	11	11	12	11	13	16	13	15	11	14	12	13	13
Neonatal disorders	14	14	11	14	10	15	15	16	16	11	13	17	12	12	16	15	12	16	13	10	20	12	14	16	13	12	19	11	12	12	15	16	15	16
Osteoarthritis	15	15	17	11	17	13	16	15	12	14	16	13	15	15	14	16	18	17	14	15	11	13	16	11	10	18	13	16	19	10	11	14	16	11
Endo/metab/blood/immune	16	16	10	17	18	16	8	14	18	16	19	15	16	17	15	18	16	18	15	11	10	10	15	15	15	11	15	17	10	18	16	10	18	17
Falls	17	18	12	16	9	22	12	17	13	17	20	20	18	16	17	22	11	12	22	20	13	18	18	17	17	8	14	18	7	19	10	19	12	19
Schizophrenia	18	17	15	18	15	17	13	18	19	18	14	16	17	18	21	17	20	8	19	16	16	17	17	18	18	19	17	9	16	15	18	15	20	18
Drug use disorders	19	20	18	19	20	21	19	19	17	19	18	19	20	20	18	20	19	20	17	18	17	19	21	22	20	20	20	19	20	20	19	18	24	15
Road injuries	20	21	19	21	19	23	21	21	20	20	17	23	23	19	20	19	17	19	23	22	21	21	19	19	19	14	22	21	17	17	20	22	21	22

C

Both sexes, All ages, 1990, YLDs per 100,000

	China	Anhui	Beijing	Chongqing	Fujian	Gansu	Guangdong	Guangxi	Guizhou	Hainan	Hebei	Heilongjiang	Henan	Hubei	Inner Mongolia	Hunan	Jiangsu	Jiangxi	Jilin	Liaoning	Ningxia	Qinghai	Shaanxi	Shandong	Shanghai	Shanxi	Sichuan	Tianjin	Tibet	Xinjiang	Yunnan	Zhejiang	Hong Kong	Macao
Low back pain	1	1	1	1	1	1	1	1	1	1	1	1	1	1	1	1	1	1	1	1	1	1	1	1	1	1	1	1	1	1	1	1	1	1
Age-related hearing loss	2	3	2	2	2	4	2	2	2	2	2	4	3	4	4	4	2	2	3	2	4	4	4	3	2	2	2	2	2	4	2	3	2	2
Depressive disorders	3	2	6	3	3	2	4	4	4	3	4	3	2	2	3	2	4	4	4	4	5	3	2	2	7	4	4	6	5	2	4	2	3	4
Headache disorders	4	4	3	4	4	3	3	3	3	4	3	2	4	3	2	5	3	3	2	3	3	2	3	4	3	3	3	3	3	3	3	4	4	3
Anxiety disorders	5	5	11	7	5	5	12	5	5	6	7	6	5	6	5	3	8	5	5	11	2	7	5	5	17	6	7	7	6	5	6	7	11	7
Gynecological diseases	6	7	4	5	7	6	5	6	6	7	6	5	7	7	6	6	6	8	6	5	6	5	8	7	4	8	5	4	9	7	5	5	7	5
Neck pain	7	8	5	6	6	7	6	7	8	8	5	7	6	8	7	7	7	7	7	6	7	6	7	6	5	7	6	5	7	8	7	8	5	6
Dietary iron deficiency	8	6	20	8	10	8	13	8	7	5	8	10	8	5	11	8	5	6	9	10	8	8	6	9	6	5	8	14	4	6	21	6	19	14
COPD	9	9	14	9	8	10	7	9	11	11	14	13	10	9	10	9	9	11	15	13	10	9	14	8	10	11	9	12	11	13	10	10	13	9
Other musculoskeletal	10	12	9	13	12	15	11	12	18	16	11	11	11	11	12	11	11	17	11	9	14	13	9	11	8	12	12	10	17	12	20	13	6	8
Diabetes	11	10	12	11	13	14	10	18	13	9	13	9	14	17	13	12	15	14	10	8	16	17	12	15	12	9	14	9	18	9	17	15	17	22
Stroke	12	14	7	19	16	20	15	17	17	12	9	8	9	14	9	16	16	18	8	7	12	18	15	12	13	10	19	8	13	16	18	18	15	10
Oral disorders	13	11	15	15	14	13	17	13	12	15	12	15	13	13	14	14	13	12	12	14	11	12	10	13	18	13	10	13	14	14	16	12	8	12
Endo/metab/blood/immune	14	15	8	16	15	17	9	14	16	14	17	14	15	12	18	15	12	16	13	12	9	11	11	14	9	17	13	11	15	15	14	11	10	11
Schizophrenia	15	16	10	18	11	16	8	19	19	17	10	12	16	15	15	20	18	9	19	15	13	15	13	10	15	15	16	15	21	19	13	14	16	13
Drug use disorders	16	18	17	14	17	9	16	10	9	18	15	16	17	16	16	10	17	10	14	16	15	10	18	17	20	14	17	16	12	11	8	17	9	15
Blindness and vision loss	17	13	16	12	18	18	14	11	21	13	16	18	12	10	17	13	14	15	17	17	18	21	16	16	19	20	20	17	8	21	12	16	12	18
Falls	18	17	13	10	9	21	18	16	14	10	20	19	20	18	23	17	10	13	18	19	21	14	17	18	11	18	18	18	10	18	15	9	14	16
Osteoarthritis	19	21	18	17	23	19	22	20	15	21	19	21	18	19	20	18	19	21	21	18	23	19	19	19	21	16	15	19	25	17	19	20	18	19
Dermatitis	20	19	23	21	20	22	24	21	22	20	18	20	19	20	19	19	20	19	20	20	17	20	20	20	14	21	21	20	22	20	22	21	20	21

D

Both sexes, All ages, 2019, YLDs per 100,000

	China	Anhui	Beijing	Chongqing	Fujian	Gansu	Guangdong	Guangxi	Guizhou	Hainan	Hebei	Heilongjiang	Henan	Hubei	Hunan	Inner Mongolia	Jiangsu	Jiangxi	Jilin	Liaoning	Ningxia	Qinghai	Shaanxi	Shandong	Shanghai	Shanxi	Sichuan	Tianjin	Tibet	Xinjiang	Yunnan	Zhejiang	Hong Kong	Macao
Age-related hearing loss	1	1	2	1	2	2	2	2	2	2	2	2	2	1	1	2	1	2	1	1	2	2	1	1	1	2	1	2	2	2	1	1	2	2
Low back pain	2	2	1	2	1	1	1	1	1	1	1	1	1	2	2	1	2	1	2	2	1	1	2	2	3	1	2	1	1	1	2	3	1	1
Headache disorders	3	3	3	4	3	4	3	3	3	4	5	3	3	4	4	3	3	3	5	5	3	3	3	4	2	3	3	4	3	3	3	4	5	3
Depressive disorders	4	4	8	3	4	3	4	4	4	5	4	6	4	3	3	4	4	4	7	6	5	4	4	3	9	5	4	8	4	4	4	2	3	5
Neck pain	5	5	5	7	5	5	6	5	5	6	7	7	6	5	6	5	5	5	8	7	6	5	5	5	5	6	6	7	5	6	5	5	6	6
Other musculoskeletal	6	8	4	9	6	8	5	6	8	7	8	8	8	6	8	6	6	6	6	8	7	6	6	6	4	7	9	6	6	7	8	6	4	4
Diabetes	7	6	6	6	7	7	7	8	7	3	6	5	7	10	7	8	8	7	4	4	8	7	8	8	6	4	7	3	11	5	6	8	10	12
Stroke	8	7	9	11	11	11	11	7	6	8	3	4	5	8	9	7	7	13	3	3	9	11	7	7	11	8	10	5	9	12	10	16	12	7
Osteoarthritis	9	13	14	8	13	10	14	12	9	13	11	9	11	11	12	10	12	16	9	9	12	9	9	13	15	9	8	13	18	9	9	15	9	9
Blindness and vision loss	10	9	13	10	12	12	12	9	14	9	9	10	9	7	11	9	10	8	10	11	14	14	11	12	16	13	14	14	7	14	11	13	8	16
Anxiety disorders	11	11	17	14	9	6	18	10	11	10	12	12	10	13	5	11	15	10	12	15	4	15	10	9	18	10	16	10	8	8	13	9	16	11
COPD	12	10	20	5	17	9	15	13	10	16	15	14	12	9	10	12	9	11	15	14	15	10	14	10	21	17	5	16	15	11	12	17	15	14
Gynecological diseases	13	14	7	15	16	13	8	11	12	11	14	15	14	12	13	15	16	15	14	13	10	8	16	16	7	12	13	11	10	10	7	11	14	8
Oral disorders	14	12	15	12	10	14	16	14	15	12	10	11	13	14	14	13	14	14	11	12	13	12	12	14	13	11	12	12	13	13	17	14	7	13
Endo/metab/blood/immune	15	15	10	16	18	15	9	15	16	14	17	13	15	16	15	16	17	17	13	10	11	13	13	17	10	18	15	9	17	15	14	10	13	10
Falls	16	17	12	13	8	21	13	16	13	18	20	17	18	15	16	20	11	12	20	19	18	17	15	18	8	19	11	17	12	21	15	7	11	17
Schizophrenia	17	16	11	17	14	17	10	17	17	17	13	16	17	18	20	17	19	9	17	16	16	16	17	11	12	14	17	15	19	17	16	18	17	15
Neonatal disorders	18	18	16	18	15	18	17	18	20	15	18	21	16	19	19	19	18	18	16	17	17	20	18	15	19	16	19	20	14	18	20	19	25	18
Road injuries	19	19	19	19	19	20	21	21	19	19	16	18	19	17	17	18	13	19	19	20	19	19	19	19	14	15	18	19	22	20	21	12	20	20
Drug use disorders	20	22	18	22	20	22	19	19	18	20	19	23	22	20	18	21	20	20	22	21	21	22	20	21	22	20	21	18	24	16	19	20	19	19

Supplementary Figure 5 Sociodemographic index (SDI) of 33 provinces/municipalities/autonomous regions in China in 1990 and 2019, and the changes between 1990 and 2019.

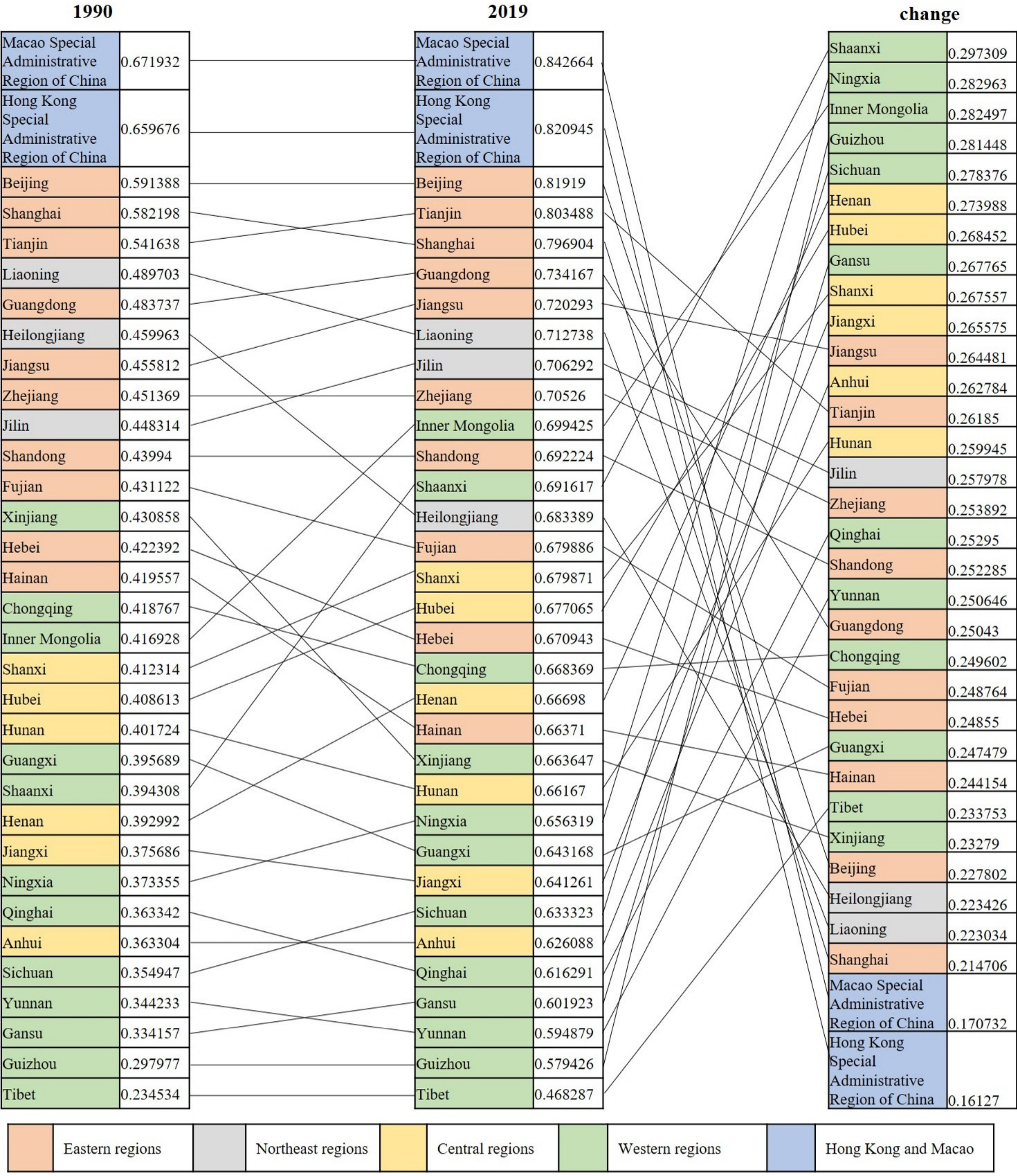

Supplementary table 1

Age-standardized incidence, prevalence and YLD rate and their changes of neck pain in 27 countries, 1990 and 2019.

Country name	Incidence (per 100000 population)		Change (%)	Prevalence (per 100000 population)		Change (%)	YLDs rate (per 100000 population)		Change (%)
	1990	2019		1990	2019		1990	2019	
**Global**	581.74 (460.94,737.68)	579.09 (457.90,729.6)	-0.46 (-2.13,1.52)	2709.42 (2172.31,3409.76)	2696.52 (2177.01,3375.19)	-0.48 (-2.58,1.67)	268.26 (176.71,382.67)	267.35 (175.53,383.54)	-0.34 (-2.13,1.52)
**Argentina**	396.75 (310.24,501.29)	396.82 (310.34,501.40)	0.02 (-0.03,0.05)	1933.69 (1539.03, 2447.46)	1934.02 (1539.41,2447.84)	0.02 (-0.02,0.05)	192.43 (125.73, 274.56)	192.24 (126.84, 273.44)	-0.10 (-2.92, 2.94)
**Australia**	246.10 (194.93, 307.43)	246.19 (194.93,307.68)	0.04 (-0.10,0.14)	1092.58 (862.77, 1384.65)	1092.71 (861.18, 1386.04)	0.01 (-0.19,0.16)	107.88 (70.51, 154.18)	107.86 (70.81, 155.14)	-0.02 (-4.03,4.19)
**Brazil**	506.19 (396.80, 645.79)	503.83 (395.14,641.96)	-0.47 (-0.70, -0.26)	2250.15 (1775.99, 2883.49)	2241.89 (1770.55, 2870.64)	-0.37 (-0.63, -0.15)	221.32 (145.14, 321.67)	221.72 (145.36, 322.57)	0.18 (-0.88,1.22)
**Burundi**	269.06 (209.48, 341.49)	266.91 (208.08,338.81)	-0.80 (-1.21, -0.51)	1154.23 (911.07, 1474.25)	1141.83 (900.81, 1458.66)	-1.07 (-1.64, -0.56)	113.91 (74.87, 164.49)	112.61 (73.96, 162.06)	-1.14 (-4.67,2.83)
**Canada**	533.86 (419.09, 675.80)	534.31 (419.47,676.66)	0.08 (0.005, 0.16)	2942.54 (2327.50, 3729.95)	2942.27 (2325.80, 3731.63)	-0.01 (-0.16,0.12)	292.87 (192.28, 423.36)	292.18 (191.39, 419.70)	-0.24 (-2.57,2.05)
**China**	800.68 (634.43, 1016.63)	809.91 (641.89,1028.61)	1.15 (-2.46,5.09)	3528.35 (2800.49, 4485.74)	3571.96 (2868.20, 4517.63)	1.24 (-4.53,7.02)	352.84 (230.02, 516.83)	358.13 (234.54, 515.95)	1.50 (-4.27,7.29)
**Denmark**	696.29 (547.19, 881.35)	696.26 (547.27, 881.31)	-0.005 (-0.10,0.06)	4486.91 (3581.42, 5617.87)	4477.65 (3571.18, 5607.69)	-0.21 (-0.36, -0.08)	446.90 (299.25, 645.48)	446.46 (298.43, 647.40)	-0.10 (-2.10,2.01)
**Djibouti**	266.49 (208.01, 338.40)	266.41 (207.64, 338.33)	-0.03 (-0.27,0.23)	1141.07 (899.44, 1457.80)	1138.49 (898.08, 1454.09)	-0.23 (-0.50,0.03)	113.49 (75.62, 163.24)	113.31 (74.08, 162.39)	-0.15 (-4.10,3.75)
**Eritrea**	269.19 (209.79, 341.71)	268.39 (209.33, 340.77)	-0.30 (-0.45, -0.19)	1156.28 (912.04, 1475.57)	1152.68 (909.12, 1471.25)	-0.31 (-0.47, -0.15)	113.50 (75.40, 163.75)	113.67 (74.48, 164.21)	0.16 (-3.74,4.10)
**Finland**	670.70 (530.65, 856.04)	671.89 (530.58, 849.84)	0.18 (-3.88,5.25)	4323.11 (3514.07, 5377.42)	4305.96 (3419.79, 5365.80)	-0.40 (-6.71,6.14)	429.80 (290.19, 628.45)	428.80 (288.49, 611.15)	-0.23 (-7.06,6.39)
**France**	507.18	507.58	0.08	2793.95	2792.68	-0.05	278.95	279.23	0.10

	(398.25, 634.25)	(398.72, 635.32)	(-0.02,0.17)	(2252.49, 3530.98)	(2250.97, 3529.56)	(-0.19, 0.07)	(187.04, 405.92)	(185.91, 406.67)	(-2.39, 2.67)
**Germany**	595.98<br>(467.90, 754.96)	594.75<br>(466.76, 753.91)	-0.21<br>(-0.43, -0.04)	3576.05<br>(2851.89,4522.22)	3552.80<br>(2833.46 4497.03)	-0.65<br>(-1.00, -0.34)	356.53<br>(233.19, 518.59)	353.80<br>(234.96, 510.13)	-0.77<br>(-3.00, 1.64)
**India**	391.37<br>(305.81, 492.84)	392.03<br>(306.34, 493.29)	0.17<br>(0.07,0.26)	1651.79<br>(1322.59, 2090.93)	1655.50<br>(1326.30, 2094.80)	0.22<br>(0.07, 0.42)	160.57<br>(107.10, 230.62)	161.95<br>(107.99, 234.14)	0.86<br>(0.15, 1.58)
**Indonesia**	965.78<br>(764.37, 1220.30)	965.00<br>(763.65, 1218.88)	-0.08<br>(-0.14, -0.02)	4306.84<br>(3413.65, 5479.71)	4302.58<br>(3410.29, 5473.63)	-0.10<br>(-0.18, 0.02)	426.01<br>(279.88, 613.84)	427.53<br>(280.32, 613.71)	0.36<br>(-0.40, 1.08)
**Italy**	774.85<br>(612.82, 975.97)	773.46<br>(611.97, 973.96)	-0.18<br>(-0.26, -0.11)	3861.70<br>(3087.74, 4893.29)	3848.98<br>(3080.42, 4874.44)	-0.33<br>(-0.47, -0.21)	383.70<br>(253.57, 545.79)	384.17<br>(254.24, 546.39)	0.12<br>(-0.60, 0.85)
**Japan**	443.92<br>(348.96, 564.14)	442.76<br>(348.05, 562.75)	-0.26<br>(-0.40, -0.17)	1962.85<br>(1550.85, 2486.90)	1954.59<br>(1544.42, 2477.85)	-0.42<br>(-0.62, -0.26)	195.82<br>(129.34, 286.66)	195.78<br>(129.81, 287.92)	-0.02<br>(-0.78, 0.65)
**Mexico**	376.96<br>(296.66, 479.95)	377.28<br>(296.90, 480.24)	0.08<br>(0.03, 0.14)	1594.04<br>(1257.94, 2056.60)	1595.89<br>(1258.92, 2058.85)	0.12<br>(0.04, 0.20)	157.47<br>(104.76, 227.83)	157.83<br>(104.51, 229.16)	0.23<br>(-0.63, 1.09)
**Norway**	658.78<br>(523.61, 833.31)	576.71<br>(453.67, 723.40)	-12.46<br>(-15.79, -8.30)	3238.82<br>(2571.94, 4127.02)	2659.99<br>(2099.64, 3366.72)	-17.87<br>(-23.67, -11.22)	321.32<br>(212.51, 465.85)	265.20<br>(174.84, 380.08)	-17.47<br>(-23.22, -10.99)
**Republic of Korea**	358.31<br>(280.32, 460.26)	356.64<br>(279.43, 458.59)	-0.47<br>(-0.66, -0.29)	1735.86<br>(1372.76, 2219.94)	1719.45<br>(1361.87, 2196.17)	-0.95<br>(-1.26, -0.59)	172.29<br>(113.62, 247.81)	171.97<br>(112.56, 247.48)	-0.19<br>(-3.33, 3.02)
**Russian Federation**	394.24<br>(308.15, 496.01)	393.79<br>(307.75, 495.45)	-0.11<br>(-0.25, -0.01)	1668.46<br>(1338.79, 2108.28)	1665.48<br>(1335.94, 2103.82)	-0.18<br>(-0.36, -0.03)	164.23<br>(108.73, 237.34)	164.90<br>(109.57, 238.37)	0.41<br>(-0.45, 1.26)
**Saudi Arabia**	578.70<br>(454.97, 738.43)	580.19<br>(455.89, 741.83)	0.26<br>(-0.06, 0.47)	2724.05<br>(2144.74, 3458.40)	2727.84<br>(2143.30, 3464.93)	0.14<br>(-0.18, 0.41)	269.06<br>(178.15, 389.13)	268.59<br>(178.87, 389.36)	-0.18<br>(-2.70, 2.42)
**South Africa**	323.08<br>(255.49, 410.96)	322.82<br>(255.27, 410.36)	-0.08<br>(-0.16, -0.01)	1365.66<br>(1077.68, 1758.11)	1364.89<br>(1077.41, 1757.51)	-0.06<br>(-0.13, 0.01)	134.50<br>(87.92, 190.86)	133.57<br>(88.16, 190.57)	-0.70<br>(-2.29, 0.84)
**South Sudan**	265.62<br>(207.36, 337.32)	268.04<br>(208.44, 340.66)	0.91<br>(0.58, 1.28)	1134.38<br>(894.88, 1447.83)	1145.76<br>(904.76, 1466.07)	1.00<br>(0.51, 1.51)	110.97<br>(73.41, 159.46)	112.12<br>(73.89, 160.59)	1.04<br>(-3.08, 5.17)
**Sweden**	760.63<br>(600.75, 956.11)	757.55<br>(598.39, 952.07)	-0.41<br>(-0.81, -0.02)	4330.89<br>(3464.19, 5479.89)	4344.75<br>(3470.51, 5510.23)	0.32<br>(-0.14, 0.91)	433.01<br>(291.64, 624.78)	434.57<br>(291.85, 628.07)	0.36<br>(-1.51, 2.14)
**Turkey**	601.94<br>(472.88, 772.22)	601.48<br>(472.59, 771.66)	-0.08<br>(-0.14, -0.03)	2875.69<br>(2253.21, 3640.54)	2875.01<br>(2252.61, 3640.76)	-0.02<br>(-0.09, 0.04)	284.85<br>(187.91, 413.80)	284.83<br>(188.62, 414.82)	-0.01<br>(-2.43, 2.48)

**United Kingdom**	827.15<br>(657.72, 1033.59)	851.18<br>(675.90, 1073.50)	2.90<br>(-1.36, 6.98)	4485.59<br>(3587.67, 5642.01)	4501.34<br>(3591.70, 5675.23)	0.35<br>(-4.98, 6.48)	443.77<br>(298.35, 641.82)	446.79<br>(301.97, 636.94)	0.68<br>(-4.84, 6.70)
**United States of America**	841.68<br>(667.47, 1068.84)	957.71<br>(771.71, 1170.78)	13.79<br>(7.68, 19.71)	4325.43<br>(3473.66, 5461.38)	5123.29<br>(4268.35, 6170.35)	18.45<br>(10.01, 27.57)	422.49<br>(279.70, 604.57)	500.26<br>(338.92, 704.86)	18.41<br>(9.89, 27.58)

Supplementary table 2. The percent of age-standardized years lived with disability (YLDs) caused by neck pain in all diseases by provinces/regions (1990 and 2019).

	1990				2019			
	**Mean (%)**	**LUI (%)**	**UUI (%)**	**Rank**	**Mean (%)**	**LUI (%)**	**UUI (%)**	**Rank**
**China**	3.71	2.87	4.81	5	4.00	3.10	5.21	5
**Anhui**	3.67	2.86	4.81	6	4.13	3.19	5.38	5
**Beijing**	3.73	2.88	4.86	6	3.92	3.02	5.09	5
**Chongqing**	3.65	2.83	4.75	6	3.94	3.04	5.11	5
**Fujian**	3.77	2.91	4.87	5	4.12	3.20	5.37	5
**Gansu**	3.57	2.76	4.63	6	3.90	3.01	5.04	6
**Guangdong**	3.70	2.85	4.83	5	3.90	3.02	5.05	6
**Guangxi**	3.68	2.85	4.82	5	4.05	3.13	5.29	5
**Guizhou**	3.47	2.68	4.53	6	3.87	2.99	5.03	5
**Hainan**	3.62	2.80	4.71	7	3.94	3.06	5.12	6
**Hebei**	3.84	2.97	4.99	5	3.99	3.09	5.20	6
**Heilongjiang**	3.62	2.78	4.74	7	3.92	3.02	5.20	7
**Henan**	3.80	2.93	4.95	5	4.08	3.15	5.31	6
**Hong Kong Special Administrative Region of China**	3.82	2.98	4.93	5	4.13	3.15	5.31	6
**Hubei**	3.69	2.82	4.79	6	4.05	3.14	5.23	5
**Hunan**	3.58	2.77	4.65	6	3.96	3.06	5.16	6
**Inner Mongolia**	3.72	2.89	4.87	5	3.98	3.06	5.15	6
**Jiangsu**	3.71	2.86	4.81	7	4.02	3.10	5.21	5
**Jiangxi**	3.64	2.81	4.73	5	4.09	3.15	5.33	5
**Jilin**	3.79	2.93	4.96	6	4.13	3.18	5.39	7

**Liaoning**	3.84	2.98	4.99	6	4.04	3.12	5.28	7
**Macao Special Administrative Region of China**	3.93	3.04	5.15	5	4.16	3.22	5.42	6
**Ningxia**	3.81	2.94	4.92	6	4.00	3.09	5.21	6
**Qinghai**	3.86	2.99	5.02	5	4.06	3.16	5.30	5
**Shaanxi**	3.86	2.98	4.99	5	4.19	3.24	5.45	5
**Shandong**	3.87	2.99	5.01	7	4.12	3.19	5.35	6
**Shanghai**	3.62	2.79	4.73	6	3.92	3.02	5.13	5
**Shanxi**	3.72	2.88	4.85	6	4.09	3.16	5.33	6
**Sichuan**	3.65	2.83	4.74	7	3.86	2.98	5.03	5
**Tianjin**	3.89	3.00	5.06	5	4.08	3.13	5.31	7
**Tibet**	3.55	2.70	4.61	7	3.89	2.98	5.04	6
**Xinjiang**	3.67	2.82	4.76	5	3.95	3.06	5.13	6
**Yunnan**	3.56	2.74	4.63	6	3.88	3.00	5.07	5
**Zhejiang**	3.63	2.78	4.72	8	3.96	3.05	5.14	5
